# *Vicia faba* L. Pod Valves: A By-Product with High Potential as an Adjuvant in the Treatment of Parkinson’s Disease

**DOI:** 10.3390/molecules29163943

**Published:** 2024-08-21

**Authors:** Carmen Tesoro, Filomena Lelario, Fabiana Piscitelli, Angela Di Capua, Paolo Della Sala, Paola Montoro, Giuliana Bianco, Maria Assunta Acquavia, Mario Dell’Agli, Stefano Piazza, Rosanna Ciriello

**Affiliations:** 1 Department of Sciences, University of Basilicata, 85100 Potenza, Italy; carmen.tesoro@unibas.it (C.T.); angela.dicapua@unibas.it (A.D.C.); giuliana.bianco@unibas.it (G.B.); maria.acquavia@unibas.it (M.A.A.); rosanna.ciriello@unibas.it (R.C.); 2 Endocannabinoid Research Group, Institute of Biomolecular Chemistry (ICB), National Research Council (CNR), 80078 Pozzuoli, Italy; fpiscitelli@icb.cnr.it; 3 Department of Chemistry and Biology, University of the Study of Salerno, 84084 Fisciano, Italy; pdellasala@unisa.it; 4 Department of Pharmacy, University of the Study of Salerno, 84084 Fisciano, Italy; pmontoro@unisa.it; 5 Department of Pharmacological and Biomolecular Sciences “Rodolfo Paoletti”, University of Milan, 20133 Milan, Italy; mario.dellagli@unimi.it (M.D.); stefano.piazza@unimi.it (S.P.)

**Keywords:** broad bean pods, L-dopa, polyphenols, natural juices, neuroprotective effect, LC-ESI/LTQ-Orbitrap/MS^2^, FT-ICR-MS

## Abstract

*Vicia faba* L. is a leguminous plant with seeds rich in nutritional compounds, such as polyphenols and L-dopa, a dopamine precursor and first-line treatment for Parkinson’s symptoms. Recently, its by-products have been revalued as a sustainable source of bioactive compounds. In this study, aqueous extracts of Lucan broad bean pod valves (BPs) were characterized to evaluate their potential use as adjuvants in severe Parkinson’s disease. L-dopa content, quantified by LC-UV, was much higher in BPs than in seeds (28.65 mg/g dw compared to 0.76 mg/g dw). In addition, vicine and convicine, the metabolites responsible for favism, were not detected in pods. LC-ESI/LTQ-Orbitrap/MS^2^ allowed the identification of the major polyphenolic compounds, including quercetin and catechin equivalents, that could ensure neuroprotection in Parkinson’s disease. ESI(±)-FT-ICR MS was used to build 2D van Krevelen diagrams; polyphenolic compounds and carbohydrates were the most representative classes. The neuroprotective activity of the extracts after MPP^+^-induced neurotoxicity in SH-SY5Y cells was also investigated. BP extracts were more effective than synthetic L-dopa, even at concentrations up to 100 µg/mL, due to the occurrence of antioxidants able to prevent oxidative stress. The stability and antioxidant component of the extracts were then emphasized by using naturally acidic solutions of *Punica granatum* L., *Ribes rubrum* L., and gooseberry (*Phyllanthus emblica* L.) as extraction solvents.

## 1. Introduction

*Vicia faba* L., commonly known as broad bean, is a species of the Fabaceae or Leguminosae family consumed worldwide in human and animal nutrition due to its low cost, ease of cultivation, and edible seeds, which contain significant amounts of carbohydrates, proteins, fibers, minerals, and secondary metabolites such as polyphenolic compounds [1,2,3]. Nowadays, it represents a substantial crop, native to Southwest Asia, North Africa, Europe, and Middle Eastern countries, and widely cultivated throughout the world [2]. In general, all pulses go through an industrial process in which the seeds are separated from the fresh legumes for canning, drying, or freezing. A mixture of leaves, stems, and empty pods (valves) results from the processing steps. The huge amount of waste products generated by the food industry poses an economic disposal issue; according to FAO (Food and Agriculture Organization) estimates, world production of *Vicia faba* L. is over 4 million tons per year [4], and this harvest reasonably generates 2.8 million tons of by-product [2]. Increased awareness of sustainable development has led to devising waste recycling strategies in the food industry, which are in line with the current trend towards zero waste technology. By-products, such as artichoke bracts and stems, products from the cocoa production process, olive oil wastewater, grape seeds and peels, and potato peels, have been identified as potential natural sources of value-added bioactive compounds [5,6,7,8,9]. Recent studies have shown that *Vicia faba* L. pods may contain bioactive compounds, including antioxidants [2,10]. Polyphenol-based diets can prevent a number of chronic diseases, such as obesity, diabetes, cancer, and heart disease [11], due to their well-known antioxidant activity [12]. Reducing the levels of increased oxidative stress through antioxidant supplementation may also be beneficial in preventing or delaying the neurodegeneration typical of Parkinson’s disease. In this respect, *Vicia faba* L. is a particularly attractive food as it contains polyphenols and high amounts of L-dopa, a bioactive compound precursor of dopamine and first-line treatment for Parkinson’s symptoms [13,14,15]. Due to the neurotoxic effect of synthetic L-dopa and its side effects, natural sources of this bioactive compound could be used as adjuvants to reduce the unpleasant effects, which include the “on-off” motor fluctuations typical of severe forms of Parkinson’s disease. Clinical studies showed that positive effects were experienced by several people with Parkinson’s disease on their Parkinson’s motor symptoms after eating cooked broad beans. They reported that their “on” time was prolonged after eating a meal of broad beans and stated that the effect was similar to that of synthetic drugs. [16]. Most of the studies reported on *Vicia faba* L. focus on the quantification of L-dopa and the characterization of antioxidants in seeds [17]. Therefore, data on the profile of bioactive compounds in plant by-products need to be strengthened.

The aim of this paper was to conduct a comparative study of the L-dopa content in Lucan broad bean pod valves (BPs) and the corresponding seeds using a previously optimized and validated LC-UV method [13], and to characterize the major polyphenolic compounds in BP aqueous extracts by a LC-ESI/LTQ-Orbitrap/MS^2^-based approach. In order to improve the environmental impact of the extraction phase, unlike the studies reported in the literature on the methanolic profile of polyphenols in *Vicia faba*, only aqueous extracts were analyzed in this paper. The nutraceutical advantages of BPs with respect to seeds are highlighted, i.e., the significantly higher content of L-dopa and the absence of vicine and convicine, metabolites responsible for the hemolytic crisis in favism patients [18]. Furthermore, the high mass accuracy, high resolution, and flexible MS/MS capability of FT-ICR mass spectrometry assisted in the acquisition of the 2D Van Krevelen diagram, which is a tool used to create molecular formula maps useful for rapid and comprehensive analysis of the more representative metabolite classes in BPs, including polyphenols. 

To obtain further confirmation for the potential use of BPs in developing a dietary supplement for patients suffering from Parkinson’s disease, the neuroprotective activity of extracts against the human neuroblastoma cell line SH-SY5Y was investigated and compared to that of synthetic L-dopa. The results are encouraging and demonstrate a significant reversible effect of the aqueous extract of Lucan BPs on cell lines, even at high concentrations, due to the synergistic effect of the antioxidant compounds that prevent the oxidative stress induced by the disease [19]. 

To increase the antioxidant content, BPs were extracted for the first time with aqueous solutions of pomegranate (*Punica granatum* L.), redcurrant (*Ribes rubrum* L.), and gooseberry (*Phyllanthus emblica* L.) [20,21,22,23]. As far as we know, there is only a report in which L-dopa extracts of *Mucuna pruriens* seeds were obtained employing *Phyllanthus emblica* solution as solvent [24]. The above plants are known to contain bioactive compounds with antioxidant, anti-inflammatory, and antiplatelet effects. Their use as a natural source of polyphenols in the development of natural supplements is well established [25,26,27]. The composition of the obtained extracts revealed an enrichment in bioactive compounds compared to pure water. Moreover, the presence of naturally occurring citric acid imparts to the extracts a degree of acidity that ensures good stability of L-dopa, with recoveries similar to those obtained with traditional and non-environmentally friendly acidification approaches. Based on these results, the use of naturally acidified juices as extraction solvents is very advantageous both from a green chemistry perspective and to ensure high stability. Furthermore, these natural extracts could be used directly to produce nutritional supplements.

## 2. Results and Discussion

### 2.1. LC/UV Quantification of L-Dopa in Lucan Broad Bean Pod Valves

**Figure 1:**
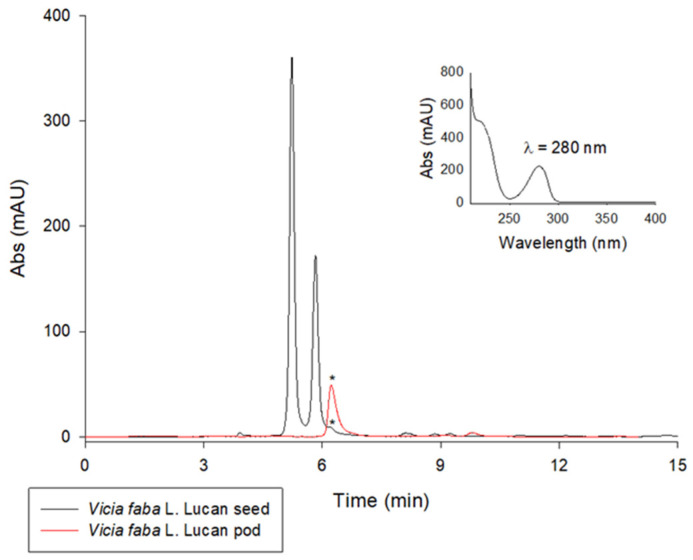
LC-UV chromatographic profiles of extracts of *Vicia faba* L. Lucan freeze-dried BPs (red plot) diluted 1:50 in 0.1 M HCl and *Vicia faba* L. Lucan freeze-dried seeds (black plot) diluted 1:10 in 0.1 M HCl. The L-dopa peak is marked with *. Discovery C_18_ column, 250 × 4.6 mm, 5 µm, was used under isocratic conditions (flow rate 1 mL/min), injection volume of 20 µL, and λ_max_ set to 280 nm.

Green chemistry is an important part of sustainable development. Waste valorization is an interesting approach that can provide several useful options for the management of food by-products other than their disposal or dumping in landfills. Therefore, this study can have a positive impact on the environment by exploring new alternatives for the valorization of *Vicia faba* by-products such as valves. Another environmentally friendly aspect of this study is the use of pure water or naturally acidic juices for extraction. This can help to reduce the use of inorganic acids as in traditional extraction methods [24]. In the first phase of this study, LC-UV analysis was carried out on aqueous extracts from freeze-dried *Vicia faba* L. Lucan BPs and seeds. A comparison of the chromatograms obtained for an acidified and an aqueous extract of *Vicia faba* L. is proposed. The LC-UV chromatograms obtained are shown in Figure 1. After appropriate dilutions in 0.1 M HCl to improve peak shape, L-dopa was quantified using the method validated by Tesoro et al. [19]. The two LC-UV chromatograms show the significant presence of L-dopa in the BP (28.65 ± 0.04 mg/g dw) compared to the seed (0.76 ± 0.11 mg/g dw). The absence of vicine and convicine in BPs is the main difference and nutraceutical advantage over the seeds, where these two toxic metabolites responsible for favism are highly concentrated. They are represented by the two main LC-UV chromatographic peaks in the black plot (Figure 1). 

### 2.2. LC-MS/MS Phenolic Compounds Characterisation in Lucan Broad Bean Pods

One of the most important families of phytochemicals found in beans is that of the phenolic compounds. These molecules play an important role in human health due to their antiviral, antimicrobial, anticarcinogenic, anti-inflammatory, and antioxidant activities [10]. While there are some publications on the polyphenol content of methanolic extracts of *Vicia faba* pods, the profile of these compounds in aqueous extracts remains to be investigated. Thus, an LC-ESI-Orbitrap-MS^2^ investigation was carried out to explore the antioxidant profile of aqueous Lucan BP extracts.

**Figure 2:**
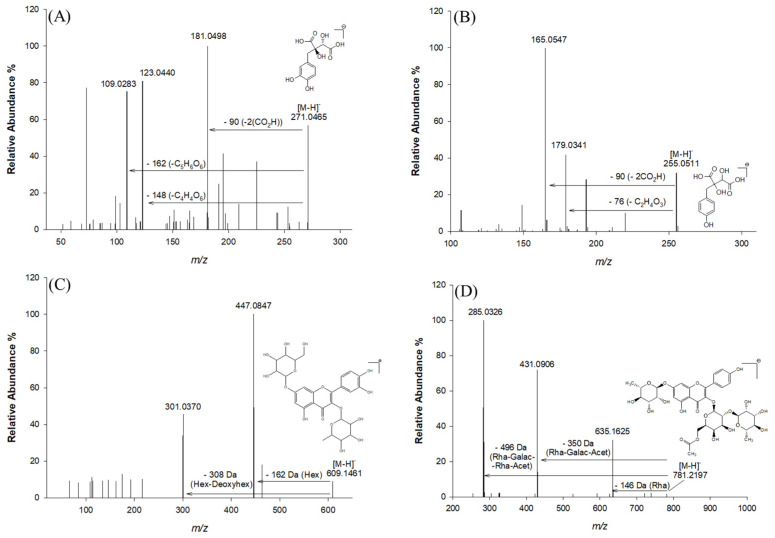
MS^2^ spectra in negative ion mode and fragmentation patterns of the most abundant compounds in the Lucan BPs: fukiic acid (**A**), piscidic acid (**B**), and a glycidic derivative of quercetin (**C**) and kaempferol (**D**).

Collisional Ion Dissociation (CID) fragmentation of the antioxidants was carried out and an unambiguous interpretation and determination of the chemical composition was obtained for most of the antioxidants (Table 1). Using data obtained from mass spectrometry (MS), tandem mass spectrometry (MS^2^), and related information previously reported in the literature, 25 major phenolic compounds were found in the extracts under investigation (Table 1). These major phenolic compounds were characterized in terms of retention time, accurate and exact *m*/*z*, mass accuracy (expressed as root mean square) [28], molecular formula generated using Xcalibur 2.0 software, and their MS^2^ fragments. The characterization was based on a comparison with what has already been reported in databases, as no commercial standards were available for most of the compounds found. A comprehensive study of the identified compounds is presented below according to their families. Glycosylated flavonoids are the most abundant phenolic compounds found in Lucan BPs, especially the glycosylated derivatives of quercetin and kaempferol, which represent two important secondary metabolites for human health as they have an anti-inflammatory effect, potential cardiovascular-related bioactivity, and a radical scavenging role. In addition, the data reported in Table 1 reveal the presence of some antioxidants characteristic of this vegetable, such as piscidic acid, quercetin hexose deoxyhexose, fukiic acid, and kaempferol-(rhamnosyl-acetyl-galactoside)-rhamnoside. Among the identified compounds, L-dopa, quercetin, and catechin equivalents have been suggested as the main components that could ensure neuroprotection in Parkinson’s disease [5]. Figure 2 shows the fragmentation pattern of the main phenolic compounds identified, i.e., fukiic acid, piscidic acid, quercetin hexose deoxyhexose, and kaempferol (rhamnosyl-acetyl-galactoside)-rhamnoside. 

#### 2.2.1. Phenolic Acids and Derivatives

Fukiic acid (compound **3**), with molecular ion at accurate *m*/*z* 271.0465 and molecular formula C_11_H_12_O_8_, was detected as one of the more representative phenolic acids in Lucan BPs, also with its methylated form methylfukiic acid (compound **6**) at accurate *m*/*z* 285.0620 (molecular formula C_12_H_14_O_8_) [2]. Other structurally similar phenolic acids have also been reported, such as piscidic acid (compound **5**), with the molecular ion at accurate *m*/*z* 255.0511 and fragment ions at *m*/*z* 193.04993, 179.03407, 165.05472, and eucomic acid (compound **8**), with a molecular ion at *m*/*z* 239.0560 [2,10]. This type of compound is characterized by MS^2^ fragments corresponding to the loss of two CO_2_H groups (−90 Da), one CO_2_H-OH group (−62 Da), and one glycolic acid residue (−76 Da) [10]. Other glycosylated phenolic acids were detected and characterized in this study. Thus, hexosides of protocatechuic acid, syringic acid, ferulic acid, coumaroylhexose, and caffeoylhexose were tentatively identified in aqueous BP extracts (compounds **4**, **2**, **11**, **7**, and **9** respectively). The MS and MS^2^ fragmentation pattern confirmed this identification since it produced the characteristic aglycone’s ions at *m*/*z* 153.0185, 197.0450, 193.0499, 163.0389, and 179.0350 after the loss of a hexose moiety [M-H-162]^−^. Coumaroylhexose (compound **7**) exhibited the diagnostic fragment at *m*/*z* 119.0489, typical of the loss of CO_2_ from a coumaroyl moiety (−44 Da) [2]. A phenolic acid linked to malic acid was also identified in this matrix; the p-coumaroyl-malic acid (compound **13**) showed the fragment corresponding to the p-coumaric acid residue (*m*/*z* 163.0390) [M-H-116]^−^, implying the loss of a C_4_H_4_O_4_ moiety attributable to the malic acid [35]. Based on MS and MS^2^ spectra and literature data, p-cumaroyl-malic acid is identified as a characteristic polyphenol of the Fabaceae family [30,36].

#### 2.2.2. Flavonoid Compounds

Glycosylated flavonoids are the most abundant phenolic compounds found in the Lucan BPs (Table 1), particularly the glycosylated derivatives of quercetin and kaempferol, which represent two important secondary metabolites for human health due to their numerous therapeutic benefits [32,37,38,39]. Compound **14** and compound **17**, having the formula C_33_H_40_O_20_ and molecular ion at *m*/*z* 755.2040, were identified as two quercetin rhamnosyl rutinoside isomers [31]. Compound **19**, characterized by a retention time of 20.06 min and a molecular ion at an accurate *m*/*z* 797.2146, was also detected. This ion had a diagnostic MS^2^ fragment at *m*/*z* 651.1551 and was tentatively identified as quercetin-rhamnosyl acetyl-hexoside-rhamnoside, due to the evident loss of a rhamnosyl moiety [M-H-146]^−^ [33]. In addition, compound **20**, with a retention time of 20.08 min and a molecular formula of C_27_H_30_O_15_, was tentatively identified as quercetin di-rhamnoside due to the loss of a rhamnosyl moiety [M-H-146]^−^ and the formation of fragment ions at *m*/*z* 447.0945 and *m*/*z* 300.9995 [10]. Kaempferol-hexoside-rhamnoside was assigned as compound **18**, characterized by a molecular ion at an accurate value of *m*/*z* 593.1512 and MS^2^ fragment ions at *m*/*z* 447.09448 and *m*/*z* 285.04056 due to the loss of rhamnosyl [M-H-146]^−^, and the loss of hexose [M-H-162]^−^ and rhamnosyl-hexose [M-H-162-146]^−^, respectively. With the precursor ion at *m*/*z* 609.1461 (compound **16**), the fragment at *m*/*z* 447.0847 appeared after the neutral loss of a hexose (162 Da). The diagnostic fragment at *m*/*z* 301.0370, which is indicative of the aglycone structure, is also of importance. Compound **16** was therefore assigned to deoxy-hexose quercetin. In addition, compound **24** also showed the diagnostic fragment ion at *m*/*z* 301.0361, corresponding to the aglycone structure of quercetin. It was therefore tentatively named quercetin acetyl rutinoside on the basis of literature data [2]. Compounds **21**, **22**, **23**, and **25** are all characterized by the presence of the diagnostic fragment ion of the aglycone kaempferol. The other fragment ions refer to losses of the sugar moieties. Specifically for compound **23**, the fragment ions at *m*/*z* 431.0906 and *m*/*z* 635.1625 are due to the loss of the rhamnosyl (−146 Da), galactosyl (−162 Da), and acetyl (−43 Da) groups and the loss of the single rhamnosyl, respectively [40].

### 2.3. Van Krevelen Plots

**Figure 3:**
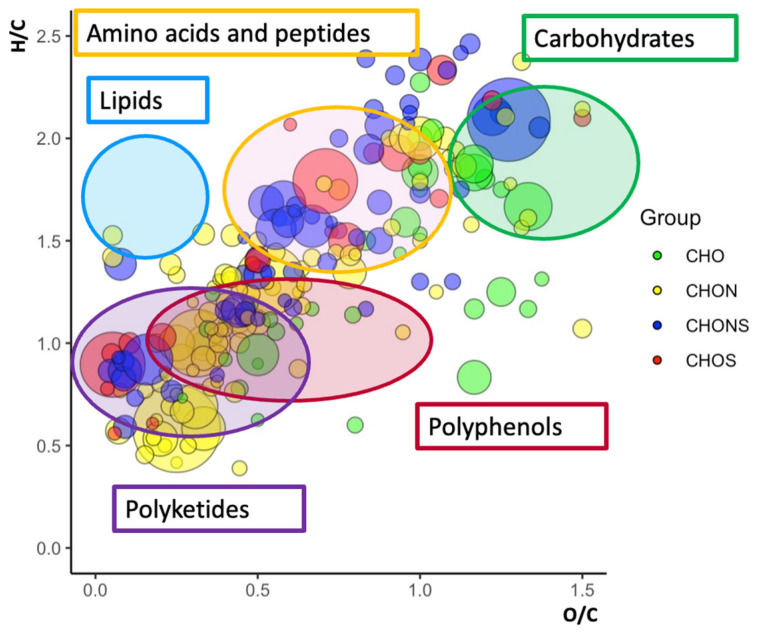
van Krevelen plots of *Vicia faba* L. aqueous pod extract obtained in negative mode from related ESI(-)-FT-ICR MS data. The type of formula is distinguished by color (green for CHO, yellow for CHON, blue for CHONS, and red for CHOS).

Ultra-high-resolution ESI(±)-FT-ICR MS data were acquired to obtain a general description of the metabolome of Lucan BP aqueous extracts. A huge number of peaks were obtained by direct injection; nearly 500 signals were selected, suggesting a wide diversity of metabolites. Interpretation of the results was facilitated by assigning MS signals to unique elementary formulas and organizing them in a well-known visualization tool i.e., the van Krevelen plot. This was employed to display our results on a 2D diagram, by setting the H/C and the O/C ratios as the *y*- and the *x*-axis, respectively (Figure 3) [41,42]. Based on H/C and O/C values, the different ionic species take up a well-defined area on the van Krevelen 2D plot. It is therefore a useful tool for classifying species into the various corresponding metabolite classes. Specifically, the lipid region is circumscribed at the highest H/C ratio (1.5 < H/C < 2.2) and lowest O/C ratio (0 < O/C < 0.5). As shown in Figure 3, the lipidic region is totally empty. The peptide region overlaps with the carbohydrate region but is located at lower O/C ratios (0.3 < O/C < 0.8) and reveals the presence of highly concentrated compounds (large circles). The carbohydrate and polyphenol regions are in the value ratio range 1.2 < H/C < 2.0, 0.6 < O/C < 1.2, and 0.6 < H/C < 1.5 and 0.4 < O/C < 1.0, respectively. A large number of signals have also been assigned in the lower part of the diagram, usually associated with polyketide compounds. The van Krevelen diagram allows us to deduce unequivocally that the *Vicia faba* L. BP aqueous extract is a rich natural matrix in terms of polyphenolic compounds, carbohydrates, and amino acid-peptides; the absence of total lipids is a key aspect that further encourages the use of this plant as a natural adjuvant in the treatment of Parkinson’s.

### 2.4. Vicia faba L. Pod Aqueous Extracts Increase Cell Viability in SHSY-5Y Cells after MPP^+^-Induced Neurotoxicity

**Figure 4:**
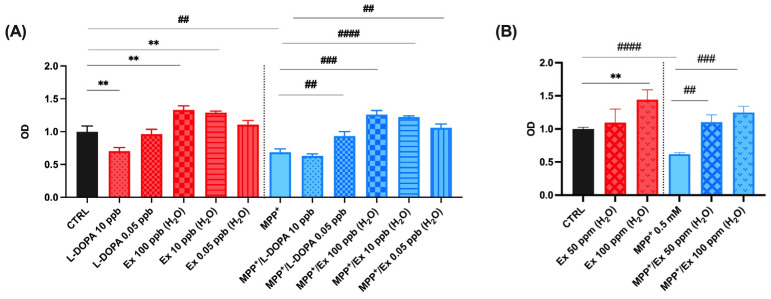
Cell viability in differentiated SH-SY5Y cells measured with the MTT assay. Histograms represent the percentage, with respect to control cells (CTRL, 100%), of viable cells after the exposure to: (**A**) MPP^+^ (0.5 mM), *Vicia faba* L. aqueous extracts 0.05, 10, 100 ng/mL (ppb) and L-dopa standard solutions 0.05, 10 ng/mL (ppb); (**B**) MPP^+^ (0.5 mM), *Vicia faba* L. aqueous extracts 50, 100 µg/mL (ppm). Data are represented as mean ± SEM (standard error of the mean). Student’s *t*-test was used for statistical analysis. The symbol # indicates values statistically significant vs. MPP^+^; the symbol * indicates values statistically significant vs. MPP^+^/L-dopa (simultaneous treatment). In particular, ** and ## indicate values statistically significant with *p* < 0.01; ### indicates values statistically significant with *p* < 0.001; #### indicates values statistically significant with *p* < 0.0001.

As shown in Figure 4A, the viability of SH-SY5Y cells treated with 0.5 mM MPP^+^ for 24 h was 61.9 ± 2.2% of the control, as previously reported [43]. The experiments were performed in triplicate and the values obtained are presented in histogram plots. Cell viability increased significantly to 93.2 ± 6.8% and 105.9 ± 5.9% after co-incubation with 0.05 ng/mL L-dopa standard and 0.05 ng/mL *Vicia faba* L. pod extracts, respectively. Differently, SH-SY5Y viability decreased for those treated after co-incubation with 10 ng/mL of L-dopa standard, characterized by a cell viability value of 63.2 ± 3.0% (Figure 4A). Those treated with up to 100 µg/mL of *Vicia faba* L. pod aqueous extracts instead maintained a cell viability of 125.0 ± 9.4% (Figure 4B). These results indicate an important nutraceutical value of the aqueous extract of *Vicia faba* L. BP, which in fact shows a greater neuroprotective effect than standard L-dopa, the use of which is limited at the cellular level, since a concentration of ≥10 ng/mL induces cell death effects.

### 2.5. L-Dopa Quantification and LC-MS/MS Antioxidant Phenolic Compound Characterization in Naturally Acidic Solutions

**Figure 5:**
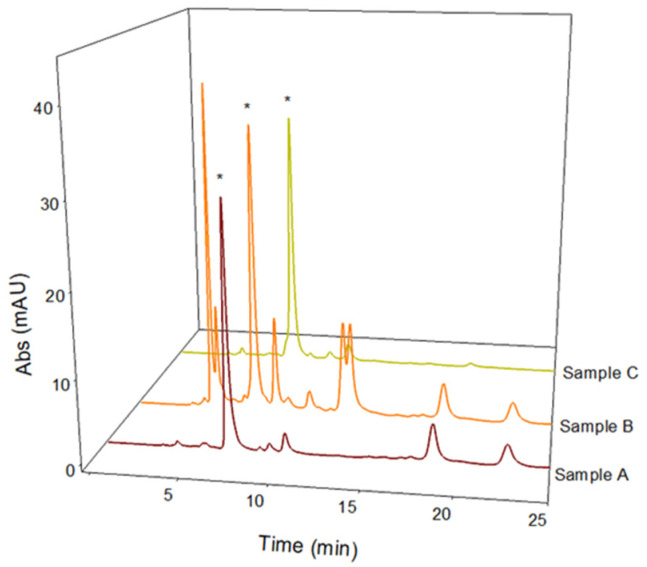
LC-UV chromatographic profiles of *Vicia faba* L. Lucan freeze-dried pods extracted in 2% *w*/*v* *Ribes rubrum* L. solution (Sample A), 2% *w*/*v* *Phyllanthus emblica* L. solution (Sample B), and 5% *w*/*v* *Punica granatum* L. solution (Sample C). The L-dopa peak is marked with *. Discovery C_18_ column, 250 × 4.6 mm, 5 µm, was used under isocratic conditions (flow rate 1 mL/min), injection volume of 20 µL, and λ_max_ set of 280 nm.

Medical research suggests that natural extracts containing high levels of L-dopa can be used successfully in the treatment of Parkinson’s disease and may reduce side effects more than the synthetic form [19]. However, these extracts are easily degraded at non-strongly acidic pH and a reduction in the L-dopa content has often been observed. An extraction method to stabilize L-dopa in aqueous extracts is therefore needed. With this aim in mind, this study investigated the extraction efficiency, stability, and antioxidant profile of three different naturally acidic fruit juices: pomegranate (*Punica granatum* L.), redcurrant (*Ribes rubrum* L.), and gooseberry (*Phyllanthus emblica* L.). The method proposed by Tesoro et al. [13] was successfully applied for the quantitative determination of L-dopa present in these natural acidic extracts of BPs. The obtained LC-UV chromatographic profiles are reported in Figure 5. Table 2 shows the amounts of L-dopa calculated in the different acidic extracts of BPs, revealing that the L-dopa content extracted using pomegranate and gooseberry juices is comparable to that extracted in pure or acidic water (recovery values of about 95%). Considering the improved stability of the extracts in acidic environments, these two natural juices can be considered good solvents for an efficient L-dopa extraction from BPs. The use of these solvents could also combine the benefits of natural matrices rich in polyphenols with the proven effect of L-dopa in the treatment of Parkinson’s disease. Therefore, the polyphenolic profile of the two potential solvents was evaluated as follows.

Regarding the major phenolic compounds, 11 were found for *Phyllanthus emblica* L., 7 for *Ribes rubrum* L., and 13 for *Punica granatum* L. (Table 3). They were tentatively characterized using mass data obtained from LC-ESI-Orbitrap-MS^2^ and the appropriate information previously reported in the literature. These compounds were characterized with respect to their retention time, accurate and exact *m*/*z*, mass accuracy (expressed as root mean square), molecular formula generated by the “Xcalibur 2.0” software and MS^2^ data fragments. A comparison with information already reported in databases was performed since no commercial standards were available for most of the compounds identified. In addition to the significant presence of important compounds, such as citric acid (which gives acidity to the extracts) and 7-(αD-glucopyranosyloxy)-2,3,4,5,6-pentahydroxyheptanoic acid, a wider presence of antioxidants in 5% *w*/*v Punica granatum* L. solution is evident. The occurrence of gallic acid, rutin, derivatives of quercetin and kaempferol, ellagic acid, and 6-*O*-(beta-D-glucopyranosyloxy)-L-ascorbic acid (derivative of ascorbic acid, i.e., vitamin C, known for its antioxidant and anti-pigmentary properties) underline the antioxidant component of the naturally acidic solutions used in the extraction of L-dopa from BPs [20,21,22,23,44]. In addition, the pH values and the percentage of L-dopa concentration calculated with respect to the value quantified in 0.1 M HCl extraction solution, especially that of *Punica granatum* L., further encourage the use of the proposed naturally acidic juices as an extracting solvent.

## 3. Materials and Methods

### 3.1. Materials

Acetonitrile (99%) was obtained from Sigma-Aldrich (Steinheim, Germany). Methanol (>99.8%) was purchased from Honeywell (Seelze, Germany). Formic acid was purchased from Fluka (Buchs, Switzerland). Ultrapure water was produced using a Milli-Q RG system from Millipore (Bedford, MA, USA). The human neuroblastoma cell line, SH-SY5Y, was purchased from American Type Culture Collection (ATCC) (Manassas, VA, USA). Dulbecco’s Modified Eagle Medium (DMEM), fetal bovine serum (FBS), penicillin and streptomycin (P/S), non-essential amino acids, phosphate-buffered saline (PBS) pH 7.4, and Trypsin/EDTA solution were purchased from GIBCO (Grand Island, NY, USA); 1-methyl-4-phenylpyridinium iodide (MPP^+^) and dimethyl sulfoxide (DMSO) from Sigma Aldrich (St. Louis, MO, USA); and Retinoic Acid (RA) from Tocris Biosciences (Bethesda, MD, USA)

### 3.2. Sample Preparation

*Vicia faba* L. broad beans were purchased from a local producer in San Chirico Raparo (Basilicata, Italy) as a fresh sample. Subsequently, the disseminated Lucan BPs were washed and freeze-dried. The extraction conditions proposed by Tesoro et al. [19] were considered with modification. Briefly, ultrasonic-assisted extraction (UAE) was carried out using an extraction ratio of 1:10 weight/dry volume and ultrapure water, HCl 0.1 M, 5% *w*/*v* (*Punica granatum* L.), redcurrant at 2% *w*/*v* (*Ribes rubrum* L.), and gooseberry at 2% *w*/*v* (*Phyllanthus emblica* L.) as extracting solutions; a sonication time of 20 min in an ice bath (4 °C) and centrifugation for 10 min at 6000× *g* were applied. This procedure was performed twice, and then supernatants were collected and filtered, first on sterile gauze and then on PTFE 0.2 µm filters. The extracts were stored at 4 °C in the dark until the LC-UV and LC-ESI/LTQ-Orbitrap/MS^2^ analyses. The L-dopa quantification on the Lucan BP extracts was performed by the external standard method, according to the validated method proposed by Tesoro et al. [13]. A botanical sample is kept in the Science Department of the University of Basilicata. The genus and species of the plant have been unambiguously identified.

### 3.3. LC-UV Conditions

The experiments were performed on an Agilent 1200 series gradient HPLC system (Agilent Technologies, Santa Clara, CA, USA) equipped with a quaternary gradient pump, a diode array detector (DAD, 190–950 nm), and a standard autosampler (0.1 µL–100 µL) set to inject 20 µL. To improve the selectivity of the method and to check the purity of the peaks, all samples were chromatographed at λ = 280 nm and the absorption spectra (190–400 nm) were recorded. All experiments were carried out at room temperature (25 °C). L-dopa was quantified using the method validated by Tesoro et al. [19] after appropriate dilutions.

### 3.4. LC-ESI/LTQ-Orbitrap/MS^2^ Conditions

The compounds in Lucan BP extracts were separated on an UltiMate 3000 Dionex UHPLC/HPLC system (Thermo Fisher Scientific, Bremen, Germany) supported with an auto-sampler and a binary, quaternary, dual-pump option. The chromatographic separation was performed in a Kinetex C_18_ analytical column (100 mm × 2.1 mm, 2.6 µm particle size). The mobile phases were formic acid 0.1%, *w*/*v* as eluent A, and acetonitrile acidified with formic acid 0.1% as eluent B. The chromatographic method comprised the following gradient: 0 min, 10% B; 20 min, 30% B; 40 min, 45% B; 60 min, 95% B; 65 min, 95% B; 75 min, 10% B. The column temperature was kept at 30 °C and the injection volume was 5 μL. The flow rate was set at 0.45 mL/min throughout the running of the gradient.

The UHPLC/HPLC system was coupled to a Q Exactive-Orbitrap (Thermo Fisher Scientific, Bremen, Germany), with an ESI interface (Agilent Technologies) operating in negative and positive ionization modes. The optimum values of the source parameters were as follows: spray voltage of ±2.5kV, spray current of 9.62 μA, capillary temperature of 300 °C, sheath gas flow rate of 50 L/min, highest energy collision dissociation (HCD) of 35 eV. The compound identification and fragmentation studies were carried out by setting full scan (in a range of *m*/*z* 120–1400) and data-dependent scan (DDA) acquisition modes. The LC-MS/MS results obtained were processed using Xcalibur version 2.0 software.

### 3.5. Van Krevelen Plots Analysis

The ESI(±) Fourier transform ion cyclotron resonance mass spectrometry (ESI-FT-ICR MS) technique was used for the untargeted analysis of the samples. High-resolution mass spectra were acquired on a Bruker (Bruker Daltonik GmbH, Bremen, Germany) solariX XR Fourier transform ion cyclotron resonance mass spectrometer (FT-ICR-MS) equipped with a 7T superconducting magnet and an ESI source. Mass spectra were acquired in negative ion mode ionization, in a mass range of 100–2000 *m*/*z*. Before the analysis, the mass spectrometer was externally calibrated with NaTFA. The FT-ICR MS data analysis was performed with Rstudio software (version 2023.12.1 + 402, w.r-project.org). Data acquisition and analysis were accomplished using the Xcalibur software package (version 2.0 SR1 Thermo Scientific). The capillary voltage was set to 3.9 and −4.5 kV for negative and positive ionization modes, respectively, with a nebulizer gas pressure of 1.2 bar and dry gas flow rate of 4 L/min at 200 °C. Samples were analyzed by direct infusion, with a syringe flow rate of 5.0 μL/h. The time-domain signal (transient) size and accumulation time were set at 8 mega-words and 0.1 s, respectively. FT-ICR mass spectra were smoothed using the Savitzky–Golay algorithm (by setting a mass range of 0.001 Da and performing 10 cycles) and exported to peak lists by considering a relative intensity threshold of 0.7% for noise filtering [19,20]. Spectra were acquired with a time-domain size of 16 mega-words and an accumulation time of 0.1 s. For each sample, the number of scans was set to 50. High accuracies were reached, with a root mean square (RMS) error lower than 0.1 ppm. To obtain unequivocal formulas, several constraints were applied, such as atom number limitations, i.e., C ≤ 100, H ≤ 200, O ≤ 80, N ≤ 5, and S ≤ 1 [41,42]; restrictions on atom to carbon number ratios, i.e., 0.2 ≤ H/C ≤ 3.1, O/C ≤ 2, N/C ≤ 1.3, and S/C ≤ 0.8, RDBE > 0; nitrogen rule (for *m*/*z* ratio values lower or equal to 500); and isotopic pattern filtering. HRMS data were processed using Data Analysis (v4.2, Bruker Daltonik GmbH, Bremen, Germany) and the R software (www.r-project.org, v3.6.3, accessed on 1 February 2024).

### 3.6. Cell Culture, Treatment, and Cell Viability Assay

Differentiated SH-SY5Y cells were maintained in DMEM supplemented with a low percentage of inactivated FBS (3% *v*/*v*) and 10 µM RA for a minimum period of 8 days. Differentiated and not cells were stored at 37 °C in a 95% humidified incubator with 5% CO_2_. The medium was changed every four days and the cells were used for no more than 25 passages. When the cells reached 80% confluence, they were detached using 0.2% (*w*/*v*) trypsin and transferred to different multi-wells according to the experimental procedure. Experiments were performed using 1.5 × 10^5^ cells/well in 24-well plates for MTT assay. Differentiated SHSY5Y cells were used first to test the cytotoxicity of 0.05, 10, and 100 ng/mL of L-dopa standard aqueous solution, and 0.05, 10, and 100 ng/mL and 50 and 100 µg/mL of Lucan BP extracts, in combination or not with 0.5 mM MPP^+^ for 24 h by quantitative colorimetric assay with MTT (0.5 mg/mL), as previously reported [43]. The extracts were added 1 h after MPP^+^ treatment. Then, the same protocol was used to test the potential neuroprotective effect of the highest non-cytotoxic concentration of L-dopa and extracts in differentiated cells 1 h after MPP^+^ treatment. After 24 h, the medium was removed from each well of the plates. Then, 300 μL of MTT reagent (0.5 mg/mL) was added and incubated in a humidified incubator at 37 °C with 5% CO_2_ for an additional 3 h period. Metabolically active cells convert the yellow MTT tetrazolium compound into a purple formazan product. The insoluble formazan was dissolved with 900 μL of isopropanol. The plates were placed on a shaker to solubilize the formations of purple crystal formazan. The absorbance was measured using a microplate reader at a wavelength of 570 nm. The results were used to construct a graph of cell viability percentage against control. Control cells treated with DMEM + Veh (methanol/ethanol < 0.03%) were taken as 100% viability [43].

## 4. Conclusions

This study confirmed that *Vicia faba* L. pod valves are rich in bioactive phytochemicals, suggesting that this legume by-product is an interesting new source of health-promoting compounds, not only for human nutrition but also for the development of new nutraceutical products. The higher content of L-dopa in pod valves compared to seeds was confirmed, with levels even comparable to those found in *Mucuna pruriens* [17]. It also highlighted the nutraceutical advantage of aqueous and naturally acidic extracts due to their increased stability, negligible content of the anti-nutritional compounds vicine and convicine, and the presence of antioxidants that could have a neuroprotective effect in Parkinson’s disease. Based on the data obtained from neuroprotective assays, the studied extracts proved to be good candidates as adjuvants in the treatment of Parkinson’s disease, as they did not show cytotoxic activity even at relatively high concentrations. Their in vivo applicability remains to be investigated in a future study.

## Figures and Tables

**Table 1:** Phenolic acids, flavonoid glycosides, and derivatives detected and characterized in Lucan *Vicia faba* L. pods.

Peak #	Retention Time (min)	Molecular Formula	^a^ *m*/*z* Exp. [M-H]^−^	^b^ *m*/*z* Calc. [M-H]^−^	^c^ Mass Accuracy (RMS)	Major Fragments	Assignment	Reference
Phenolic acid derivatives								
1	0.60	C_9_H_8_O_4_	179.0343	179.0349	0.44	134.9868	Caffeic acid	[28]
2	0.90	C_15_H_20_O_10_	359.0990	359.0972	1.84	197.0450	Syringic acid hexoside	[2]
3	0.91	C_11_H_12_O_8_	271.0465	271.0459	1.99	181.0498; 123.0440; 109.0283; 165.0546; 151.0392	Fukiic acid	[2]
4	1.24	C_13_H_16_O_9_	315.0727	315.0722	1.60	152.0105; 153.0185; 108.0208; 109.0282	Protocatechuic acid hexoside	[29]
5	1.56	C_11_H_12_O_7_	255.0511	255.0510	0.17	193.0499; 179.0341; 165.0547	Piscidic acid	[2]
6	3.47	C_12_H_14_O_8_	285.0620	285.0616	1.47	209.0458; 195.0655;	Methylfukiic acid	[2]
7	3.80	C_15_H_18_O_8_	325.0930	325.0929	0.31	163.0389; 119.0489	Coumaroylhexose	[29]
8	4.57	C_11_H_12_O_6_	239.0560	239.0561	0.38	178.9771	Eucomic acid	[29]
9	6.29	C_15_H_18_O_9_	341.0881	341.0878	0.95	179.0350	Caffeoylhexose	[2]
10	6.48	C_19_H_22_O_13_	457.0991	457.0988	0.85	179.0340	Cutaric acid hexoside	[2]
11	6.61	C_16_H_20_O_9_	355.1042	355.1035	1.98	193.0499	Ferulic acid hexoside	[2]
12	9.11	C_13_H_12_O_8_	295.0458	295.0459	0.65	133.0131; 115.0023; 135.0440	Coutaric acid or Phaseolic acid	[2]
13	12.07	C_13_H_12_O_7_	279.0513	279.0510	0.98	163.0390; 119.0492	*p*-Coumaroyl-malic acid	[30]
Flavonoid derivatives								
14	13.41	C_33_H_40_O_20_	755.2040	755.2049	1.25	301.0449; 447.0854	Quercetin rhamnosyl rutinoside	[31]
15	15.55	C_33_H_40_O_19_	739.2112	739.2091	2.89	431.0901; 593.1484	Rhoifolin glucoside	[32]
16	17.58	C_27_H_30_O_16_	609.1461	609.1474	2.12	447.0847; 301.0370	Quercetin hexose deoxyhexose	[29]
17	18.77	C_33_H_40_O_20_	755.2058	755.2040	2.30	301.0349	Quercetin rhamnosylrutinoside	[2]
18	20.02	C_27_H_30_O_15_	593.1512	593.1522	1.73	447.0945; 285.0406;	Kaempferol-hexoside-rhamnoside	[32]
19	20.06	C_35_H_42_O_21_	797.2146	797.2166	2.47	651.1550; 447.0854	Quercetin-rhamnosyl acetyl-hexoside-rhamnoside	[33]
20	20.08	C_27_H_30_O_15_	593.1512	593.1525	2.15	447.0945; 300.9995	Quercetin di-rhamnoside	[2]
21	20.11	C_27_H_30_O_15_	593.1524	593.1512	2.03	285.0406; 284.0306	Kaempferol 3-*O*-rutinoside	[34]
22	20.97	C_33_H_40_O_19_	739.2091	739.2112	2.89	285.0406; 593.1508	Kaempferol-rhamnosyl-galactoside-rhamnoside	[2]
23	21.58	C_35_H_42_O_20_	781.2197	781.2219	2.81	635.1625; 431.0906; 285.0426	Kaempferol-(rhamnosyl-acetyl-galactoside)-rhamnoside	[32]
24	22.09	C_29_H_32_O_17_	651.1567	651.1582	2.35	301.0361; 447.0853	Quercetin-acetyl-rutinoside	[2]
25	24.84	C_29_H_32_O_16_	635.1618	635.1621	0.55	285.0405; 489.1048; 431.0906	Kaempferol-acetyl-glucoside-rhamnoside	[32]

^a^ average value of *n* = 5 *m*/*z* measurements; ^b^ the exact masses were calculated by using Xcalibur software (2.0 Thermo Fisher Scientific, Bremen, Germany); ^c^ mass accuracies expressed as root mean square (RMS) in parts per million (ppm) of five (*n* = 5) *m*/*z* measurements. Parts per million: (accurate mass − exact mass) × 10^6^/exact mass.

**Table 2:** L-dopa content in naturally acidic *Vicia faba* L. BP extracts, expressed as (mg/g dw) ± calibration fitting error (mg/g). Values marked by the same letter are not significantly different (*p* < 0.05).

L-Dopa (mg/g dw) in 2% *w*/*v Phyllanthus emblica* L. BP Extract	L-Dopa (mg/g dw) in 5% *w*/*v Punica granatum* L. BP Extract	L-Dopa (mg/g dw) in 2% *w*/*v Ribes rubrum* L. BP Extract	L-Dopa (mg/g dw) in HCl 0.1 M BP Extract	% L-Dopa (mg/g dw) in Naturally Acidic Aqueous Solutions BP Extracts/L-Dopa (mg/g dw) in HCl 0.1 M BPs Extract	pH Value of 2% *w*/*v Phyllanthus emblica* L. Solution	pH Value of 5% *w*/*v Punica granatum* L. Solution	pH Value of 2% *w*/*v Ribes rubrum* L. Solution
21.87 ± 0.76 (a)	21.84 ± 0.75 (a)	16.65 ± 0.80 (b)	22.95 ± 0.74 (a)	95% in 2% *w*/*v* *Phyllanthus emblica* L. 95% in 5% *w*/*v* *Punica granatum* L. 73% in 2% *w*/*v* *Ribes rubrum* L.	3.30	3.79	3.37

**Table 3:** Phenolic acids, flavonoid glycosides, and derivatives detected and characterized in Lucan *Vicia faba* L. pods extracted in 2% *w*/*v Phyllanthus emblica* L., 5% *w*/*v Punica granatum* L., and 2% *w*/*v Ribes rubrum* L.

*Phyllanthus emblica* L.	*Ribes rubrum* L.	*Punica granatum* L.	Ion Form	*m*/*z* Calc. ^a^	*m*/*z* Exp. ^b^	Molecular Formula	Mass Accuracy (RMS) ^c^	Major Fragments	Assignment	Reference
yes	no	yes	[M-H]^−^	169.0142	169.0140	C_7_H_6_O_5_	1.3	125.0246; 169.0142	Gallic Acid	[45]
yes	yes	yes	[M+H]^+^	449.1078	449.1066	C_21_H_20_O_11_	2.8	287.0543	Kaempferol-3-*O*-glucoside	[46]
yes	yes	yes	[M-H]^−^	463.0882	463.0871	C_21_H_20_O_12_	2.4	300.9982	Quercetin 3-*O*-hexoside	[46,47]
no	yes	yes	[M+H]^+^	611.1607	611.1585	C_27_H_30_O_16_	3.3	449.1068; 287.0544	Rutin	[46]
yes	no	yes	[M-H]^−^	355.1035	355.1021	C_16_H_20_O_9_	3.7	179.0558; 193.0350; 161.0453	Ferulic acid 4-*O*-glucoside	[48]
yes	no	yes	[M-H]^−^	447.0569	447.0556	C_20_H_16_O_12_	2.8	300.9986	Ellagic acid deoxyhexoside	[46]
yes	no	yes	[M-H]^−^	633.0733	633.0706	C_27_H_22_O_18_	4.4	300.9990; 275.0197; 615.0624	Phyllanemblinin B	[47]
yes	no	yes	[M-H]^−^	300.9989	300.9982	C_14_H_6_O_8_	2.7	257.0089; 229.0140; 185.0243	Ellagic acid	[49]
yes	no	yes	[M-H]^−^	447.0569	447.0556	C_20_H_16_O_12_	2.8	299.9912; 300.9986	Ellagic acid-rhamnopyranoside	[50]
yes	yes	yes	[M-H]^−^	191.0197	191.0196	C_6_H_8_O_7_	0.76	111.0090	Citric acid	[51]
yes	yes	yes	[M-H]^−^	331.0671	331.0664	C_13_H_16_O_10_	2.17	169.0143; 271.0459; 211.0248	Galloylglucose isomer I/II	[50]
no	yes	no	[M-H]^−^	477.1038	nd ^d^	C_22_H_22_O_12_		301.0345; 112.9855; 174.9559; 85.0769	Hesperetin 30-*O*-glucuronide	[48]
yes	yes	yes	[M-H]^−^	515.1195	515.1222	C_25_H_24_O_12_	5.22	353.0717; 191.0195; 179.0559	1,5-Dicaffeoylquinic acid	[48]
no	yes	yes	[M-H]^−^	353.0725	353.0718	C_12_H_18_O_12_	2.26	173.0092;111.0090	6-*O*-(beta-D-glucopyranosyloxy)-L-ascorbic acid	[52]
no	yes	yes	[M-H]^−^	387.1144	387.1126	C_13_H_24_O_13_	4.7	225.0612;179.0559;89.0247;161.0454	7-(αD-glucopyranosyloxy)-2,3,4,5,6-pentahydroxyheptanoic acid.	[53]
yes	no	yes	[M-H]^−^	633.0727	633.0706	C_27_H_21_O_18_	4.39	300.9990;275.0197;463.0515	Phyllanemblinin B	[47]

^a^ average value of *n* = 5 *m*/*z* measurements; ^b^ the exact masses were calculated using Xcalibur software (2.0 Thermo Fisher Scientific, Bremen, Germany); ^c^ mass accuracies expressed as root mean square (RMS) in parts per million (ppm) of five (*n* = 5) *m*/*z* measurements. Parts per million: (accurate mass − exact mass) × 10^6^/exact mass. ^d^ not detected.

## Data Availability

Data supporting reported results are available from the authors.
